# Understanding the Tensile Deformation Behavior of a Serviced 304 Stainless Steel Based on Quasi In Situ EBSD Measurement

**DOI:** 10.3390/ma19143146

**Published:** 2026-07-22

**Authors:** Daicun Ding, Zhijin Ji, Yan Jing, Shilong Xing, Guanghua Yan, Shuo Wu, Lingkun Zhang

**Affiliations:** 1 School of Construction Machinery, Shandong Jiaotong University, Jinan 250357, China; 206008@sdjtu.edu.cn (D.D.); 206019@sdjtu.edu.cn (Y.J.); 240012@sdjtu.edu.cn (S.X.); 206069@sdjtu.edu.cn (G.Y.); 2 Trier College of Sustainable Technology, Yantai University, Yantai 264005, China; jizhijin@s.ytu.edu.cn

**Keywords:** strain hardening, mechanical twins, strain induced martensite, EBSD measurement

## Abstract

The microstructural evolution and strain-hardening mechanisms of a serviced 304 stainless steel during tensile deformation are investigated using quasi in situ EBSD measurements. This steel exhibits a high ultimate tensile strength of about 652 MPa alongside an exceptional fracture elongation of 83.4%. Its strain hardening behavior can be divided into three distinct stages. Deformation induces heterogeneous lattice rotation, which is dominated by the preferential activation of slip systems with the top two Schmid factors. With increasing strain, the deformation mechanism evolves sequentially from dislocation slip to mechanical twinning and then strain-induced martensite transformation. Mechanical twins act as the preferential nucleation sites for strain-induced martensite. In the latter two deformation stages, mechanical twinning serves as the primary driver of strain hardening, while strain-induced martensite merely contributes auxiliary hardening due to its limited volume fraction. This work elucidates the full-chain deformation mechanism of serviced 304 stainless steel. It provides experimental fundamentals for evaluating the residual ductility and failure risk of serviced austenitic stainless steel components.

## 1. Introduction

The microstructural evolution during plastic deformation remains a central topic in materials engineering research. It is particularly true for metastable austenitic stainless steels, the service performance of which strongly depends on deformation-driven microstructural responses. The plastic deformation of these alloys generates stacking faults, mechanical twins and martensite [1,2,3], alongside dislocation multiplication, substructure formation, lattice rotation and grain refinement [4,5,6,7,8]. These microstructural changes correspond to different deformation mechanisms. The synergistic interactions between these deformation mechanisms endow the material with high strength, remarkable ductility and good formability [9,10]. The transition of deformation mechanisms during the deformation process produces multi-stage strain hardening behavior in metastable austenitic stainless steels [11,12,13]. Investigating these evolution mechanisms is of paramount importance for both scientific understanding and industrial application.

As a typical metastable austenitic stainless steel, 304 stainless steel is widely used in the chemical, medical, energy, and aerospace industries, due to its outstanding mechanical properties and exceptional corrosion resistance [14,15,16]. Extensive studies have been conducted on the plastic deformation mechanisms of this steel at both room and low temperatures [17,18,19]. Dislocation glide, martensite transformation, and twinning are recognized as the key deformation mechanisms. Dislocation glide on activated slip systems can be considered the fundamental driving force for the occurrence of aforementioned deformation mechanisms. Therefore, identifying the activated slip systems is a critical first step in analyzing microstructural evolution. Compared with other characterization techniques, electron backscatter diffraction (EBSD) can provide more comprehensive information, such as grain orientation, crystallographic texture, and deformation substructures, enabling the determination of activated slip systems [20,21]. Combined with interrupted tensile testing, this approach enables in situ or quasi in situ characterization of specific sample regions at different strain levels, allowing for the systematic monitoring of microstructural evolution during plastic deformation [22,23,24]. Then, the active deformation mechanisms and the underlying strengthening principles can be elucidated based on these results.

Most existing investigations focus on the deformation behaviors of annealed 304 stainless steel [17,18,19,25]. Nevertheless, relevant knowledge about serviced counterparts is still insufficient. The deformation behaviors observed in annealed samples cannot reliably predict the service safety of the serviced structural components in actual service environments. Therefore, it is essential to clarify differences in deformation mechanisms between serviced and annealed 304 stainless steel. In this work, the microstructural evolution, including lattice rotation, activation of the slip system, dislocation accumulation, mechanical twins and martensite transformation, in a serviced 304 stainless steel during tensile deformation is characterized using quasi in situ EBSD measurement. By correlating microstructural evolution with mechanical response, the active deformation mechanisms and corresponding strengthening principles are explored for this steel. These results deepen the fundamental understanding of strain hardening in metastable austenitic stainless steels and supply experimental evidence for the residual ductility and failure risk assessment of serviced 304 based structural components.

## 2. Experimental Procedure

**Figure 1:**
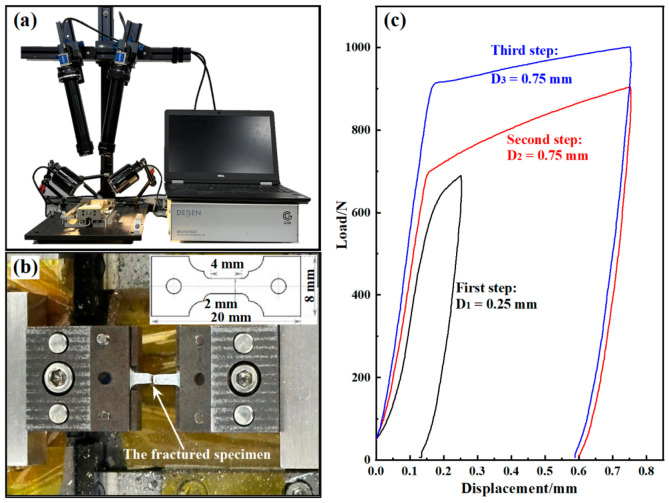
(**a**) The tensile device, (**b**) the geometry of the tensile specimen, and (**c**) the load–displacement curves of the three steps for quasi in situ tensile tests.

The experimental material was taken from the crossfire tube of a heavy-duty gas turbine that had been in service for over 100,000 h. Long-term meta-wall temperatures of the crossfire tube ranged from 700 to 850 °C. The chemical composition of the steel was determined as Fe-0.04C-19.75Cr-7.66Ni-1.47Mn-0.37Si-0.30Mo (wt.%) using an optical emission spectrometer (Thermo Fisher Scientific ARL 4460, Thermo Fisher Scientific, Waltham, MA, USA). The stacking fault energy (SFE) was determined to be approximately 15.8 mJ/m^2^, employing the formula proposed in the literature [26]. The initial microstructures of the material were characterized by EBSD measurement, which was executed using a field emission scanning electron microscope (SEM) (TESCAN LYRA3, TESCAN ORSAY HOLDING, Brno, Czech Republics) at an accelerating voltage of 20 kV, a working distance of 9 mm and a step size of 0.5 µm. The sample surface for EBSD characterization was processed by a nano-chemical mechanical polisher (Buehler EcoMet 250, Buehler, Lake Bluff, IL, USA) through sequential conventional mechanical grinding, mechanical polishing and chemical polishing with 50 nm colloidal silica particles. To obtain the tensile properties of the steel, tensile tests with a tensile speed of 0.1 mm/min were performed on a tensile device with a loading system of GATAN MTEST 5000W (Gatan, Inc., Pleasanton, CA, USA), as shown in Figure 1a. Flat uniaxial tensile specimens with gauge dimensions of a 2 mm width, 0.8 mm thickness and 4 mm length were machined from the as-received material along the axial direction of the crossfire tube, as shown in Figure 1b.

**Figure 2:**
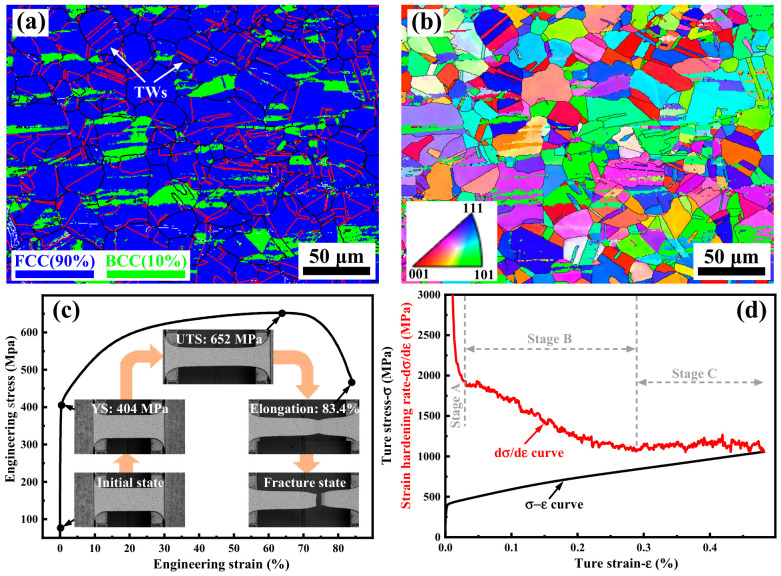
Characteristics of the as-received material, (**a**) EBSD phase map of the microstructure, (**b**) EBSD orientation map of the microstructure, (**c**) engineering stress–strain curve, and (**d**) true stress–strain curve and corresponding strain hardening rate curve.

To perform quasi in situ EBSD measurements, the tensile specimen underwent a sequential workflow: surface preparation, EBSD scanning of the marked region, tensile loading and unloading, and repeated EBSD scanning on the identical area. The interrupted tensile tests were also conducted on the tensile device by controlling the displacement. When the displacement reached a certain value, the tensile test was unloaded for quasi in situ EBSD measurements. As shown in Figure 1c, the displacements in the first, second and third deformation steps (D_1_, D_2_ and D_3_) were 0.25, 0.75 and 0.75 mm, respectively. The displacement applied at each deformation step was determined according to the true stress–strain curve and the corresponding strain hardening rate curve shown in Figure 2d. The accumulated true strains of the tensile specimen after deformation steps D_1_, D_2_ and D_3_ were approximately 0.04, 0.23 and 0.33. These values correspond to the early and middle-late periods of Stage B and the initiation of Stage C on the strain hardening rate curve, respectively. The parameters adopted for the quasi in situ EBSD measurements were consistent with those used for the initial microstructure characterization, while the step size was reduced to 0.1 μm to enable detailed characterization of the micro-regions. The EBSD datasets were post-processed using AZtecCrystal 2.1 software.

## 3. Results and Discussions

### 3.1. Initial Structural and Tensile Characteristics

Figure 2 shows the microstructure and mechanical properties of the as-received material. The EBSD phase map in Figure 2a reveals a dual-phase microstructure consisting of FCC-structured austenite (90 vol.%) and BCC-structured martensite (10 vol.%). The austenite consists of equiaxed grains characterized by an average size of 17.04 μm and random crystallographic orientations, as confirmed by the EBSD orientation map in Figure 2b. Numerous twins are observed in the austenite grains. As shown in Figure 2a, the twin boundaries (TWs) are highlighted in red lines.

The typical engineering stress–strain curve and the macro-morphologies of the tensile specimen are shown in Figure 2c. No yield plateau is observed in the tensile curve, demonstrating a smooth elasto-plastic transition. The yield strength (YS) and ultimate tensile strength (UTS) are determined to be about 404 MPa and 652 MPa, respectively. The steel exhibits excellent ductility, characterized by a fracture elongation as high as 83.4% and a uniform elongation exceeding 60%. Figure 2d depicts the true stress–strain curve (σ-ε) and corresponding strain hardening rate curve. The strain hardening rate (dσ/dε) is determined by computing the first derivative of the true stress with respect to the true strain. Three distinct stages of strain hardening, labeled as A, B and C, are clearly indicated in the dσ/dε curve. In stage A, the strain hardening rate decreases rapidly within a true strain range of less than 0.028. In stage B, the strain hardening rate exhibits a gradual deceleration in its decline in the true strain interval of 0.028–0.288. In stage C, the strain hardening rate maintains at a constant value of about 1130 MPa. At a true strain of 0.482, the true stress equals the strain hardening rate, with the intersection point indicating the onset of necking [27,28]. The different stages of strain hardening behavior are generally related to the activation of multiple deformation mechanisms. At the early stage of plastic deformation (Stage A), dislocation slip serves as the predominant deformation mechanism. The resultant dislocation accumulation creates strong barriers to further dislocation motion, thus leading to strain hardening. The rapid decrease in strain hardening rate observed in this stage is commonly attributed to the competing effect of dynamic recovery [29,30]. The strain hardening behavior in stages B and C indicates the activation of secondary deformation mechanisms, such as mechanical twinning and/or strain-induced martensite transformation, which commonly develop in alloys with low SFE during plastic deformation [31,32]. Therefore, the microstructural evolution during quasi in situ tensile deformation is analyzed in the following sections to reveal the dominant deformation mechanisms in stages B and C.

### 3.2. Slip Activity and Lattice Rotation During Deformation

**Figure 3:**
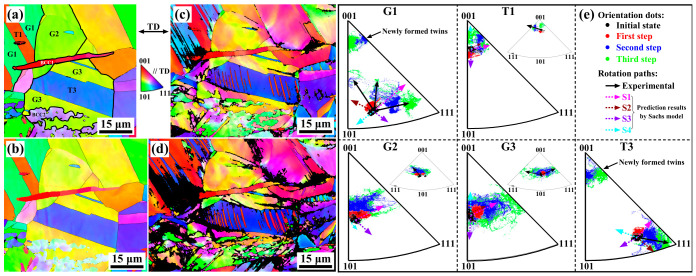
Grain orientation maps of a randomly selected region at different stages of deformation: (**a**) initial state, (**b**) first step, (**c**) second step, and (**d**) third step. (**e**) Orientation evolution of certain grains shown in IPF.

Figure 3a–d presents the grain orientation maps of a randomly selected region on the tensile specimen at different deformation steps. The plate’s normal direction corresponds to the viewing direction, while the horizontal direction is defined as the tensile direction (TD) in these maps. Adjacent pixels with a misorientation of less than 10° are defined as constituting a single grain in the undeformed sample. To trace the microstructural evolution, several representative grains are selected and marked in Figure 3a as G1, G2, and G3. The twins observed in G1 and G3 are further designated as T1 and T3, respectively. Prior to the deformation, the uniformity in color within each grain indicates a homogeneous initial crystallographic orientation. Most grains exhibit an average misorientation below 1°, whereas G2 has a relatively large average misorientation value of 2.58°. Throughout the deformation process, the development of color gradients in some grains signifies the formation of intragranular orientation gradients. As a result, the average misorientation of individual grains therefore shows a strain-dependent increase. Taking G2 as an example, the average misorientation increases to 4.63° and 6.16° after the second and third deformation steps, respectively. The change in grain orientation indicates the occurrence of lattice rotation, which is closely related to the dislocation slip in the slip system. The rotation path is considered an identifier of active slip systems, with the specific slip system leading to a distinct rotation path [8]. Therefore, the analysis of the rotation path can be used to determine the active slip systems for a given grain.

The evolution of grain orientation parallel to the TD during deformation is analyzed in the inverse pole figure (IPF) for the selected grains, G1, G2, G3, T1 and T3, as shown in Figure 3e. For an individual grain, the experimentally measured rotation paths are displayed as a sequence of colored dots (black → red → blue → green) and are denoted by solid black vectors. It can be seen that lattice rotation occurs continuously in these grains during the deformation process, resulting in an increasing deviation from the [101] orientation with respect to the TD. This result is consistent with findings reported for other FCC-structured alloys [33,34,35]. G1 and G3 have two or more rotation paths, with different regions rotating in distinct directions. This leads to the formation of subregions with differing orientations, thereby confirming the inhomogeneous deformation within the grains. T1 and T3 follow single primary rotation paths with minimal local misorientation after deformation. By contrast, a large internal misorientation and deformation substructure develop in the deformed G2, despite it also having only one primary rotation path. Careful observation of the deformed G2 in Figure 2c,d reveals a discernible orientation evolution in part of the grain, while a minority of G2 remains orientation-stable. Therefore, it can be deduced that the large internal misorientation in the deformed G2 results from the strain accommodation of heterogeneous deformation, which is primarily concentrated at the interface of rotated and stable regions.

The activation of slip systems is primarily governed by their Schmid factors, which quantifies the resolved shear stress acting on a specific slip system under applied macroscopic loading and determines the ease of dislocation slip activation. To identify the most potentially active systems, the Schmid factors of all 12 FCC slip systems are calculated for the selected grains, following established methods [33,36]. For each grain, these slip systems are ranked in descending order of their Schmid factors, with the top four systems (S1, S2, S3, and S4) and their corresponding Schmid factors listed in Table 1. The theoretical rotation paths caused by the activation of an individual slip system are calculated using the Sachs model [36,37]. The pink, brown, purple and cyan dotted arrows in Figure 3e represent the theoretical rotation paths caused by the activation of S1, S2, S3, and S4, respectively. These computed paths are then compared with the experimentally measured rotation paths in Figure 3e. The slip system that exhibits a calculated path matching the experimentally measured rotation path is identified as the active one. Accordingly, the active slip systems are identified as S2 for T1 and G2, and S1 and S2 for G3. It is typical for the experimentally measured rotation path observed to result from the activation of multiple slip systems. For example, the active slip systems are identified as S1–S3 for G1 and S1–S2 for T3. The slip systems with maximum and secondary Schmid factors (S1 and S2) are often activated first. However, slip system S3 is also activated in G1 due to its comparable Schmid factor to that of S2. It can be inferred that the plastic deformation is mainly governed by the activation of the slip system with a high Schmid factor (>0.4). To coordinate deformation, other slip systems with a small Schmid factor (<0.4) can also be activated when the local stress caused by inhomogeneous deformation exceeds the critical resolved shear stress for activating the slip system, especially in the regions near the grain boundaries [38].

**Figure 4:**
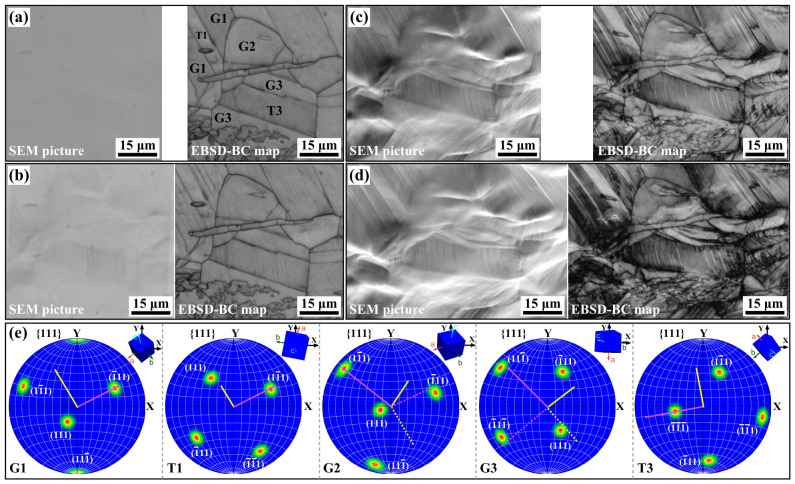
SEM images and EBSD-BC maps of the randomly selected region at different stages of deformation, (**a**) initial state, (**b**) first step, (**c**) second step, and (**d**) third step, and (**e**) {111} pole figures of certain grains in the initial state.

SEM pictures and EBSD band contrast (BC) maps of the region of interest are shown in Figure 4a–d. Surface fluctuations and slip traces are clearly observed on the tensile sample. These features offer direct evidence of grain rotation and dislocation glide. The FCC-structured grain features only four slip planes and exhibits high symmetry [36]. Therefore, the slip traces of different slip planes are clearly distinguishable on the deformed sample surface. The slip trace is the line of intersection between a slip plane and the sample surface. Combined with the {111} pole figure of a given grain, analyzing the slip traces that appear within it allows for accurate identification of the active slip plane. The slip systems containing this slip plane and those possessing the largest Schmid factor should be the ones that are activated. Figure 4e shows the {111} pole figures of the selected grains. Experimentally measured {111} slip traces are represented by yellow lines, while pink lines indicate the normals to the {111} planes. Based on slip trace analysis, the active slip systems are S1 for G1, S2 for T1, S1 and S2 for G2, S1 and S2 for G3, and S1 or S2 for T3. These results are consistent with those from the rotation path analysis. Moreover, the slip traces in G2 and G3 are less distinct than those in G1, T1, and T3, suggesting that dislocation motion was significantly inhibited in the former two grains. The most plausible explanation is that this arises from the strengthening effect of the adjacent BCC grains.

### 3.3. Deformation Heterogeneity and Substructure

**Figure 5:**
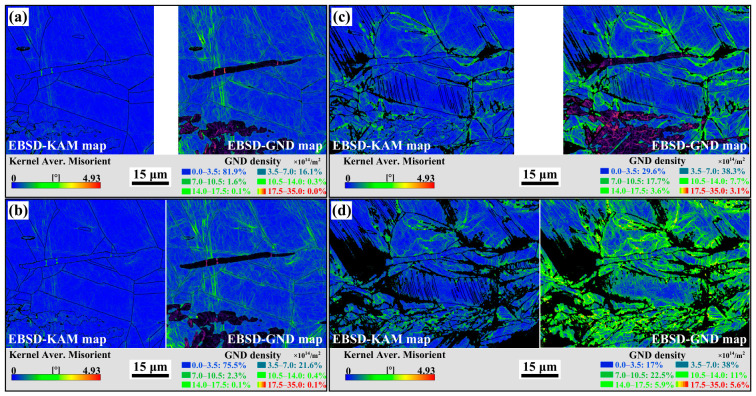
EBSD-KAM maps and GND maps of the randomly selected region at different stages of deformation: (**a**) initial state, (**b**) first step, (**c**) second step, and (**d**) third step.

Kernel average misorientation (KAM) quantifies average misorientation between individual pixels and adjacent neighbors. Given that intragranular misorientation stems from the accumulation of geometrically necessary dislocation (GND), KAM values and GND densities can therefore be used to evaluate the local strain distributions and development of substructures [39,40]. Figure 5 presents the KAM maps and the corresponding GND density maps for the selected region after different deformation steps, with the GND density values divided into six discrete ranges. The regions of high KAM values coincide with areas of high GND density. It is evident that the regions of high KAM values expand with progressive deformation. Meanwhile, the amount of GND stored in the deformed microstructure also undergo an increase, particularly during the second and third deformation steps. The first step, characterized by limited plastic deformation, only results in a modest increase in the proportion of GND densities above 3.5 × 10^14^/m^2^ (from 18.1% to 24.5%). This proportion increases sharply to 70.4% after the second step and further to 83% following the third step. It should be noted that the degradation of diffraction pattern quality in these later steps reduced the number of data points available for GND calculations.

The distribution of the KAM value and GND density became increasingly heterogeneous as deformation proceeded, indicating strain localization. Elevated KAM values and GND densities were predominantly found at regions of high lattice misfit or strain incompatibility, such as grain boundaries, austenite–martensite interfaces, and shear bands. Thus indicates that these sites experience greater local strain. Adjacent austenite grains activate distinct slip systems and follow separate rotation paths. To accommodate the strain gradient, GNDs evolve to impose plastic compatibility near the boundaries of these deforming grains [41]. Within individual grains, regions adjacent to grain boundaries often undergo rotation paths that differ from those in the grain interior. This localized heterogeneity leads to the development of steep gradients in both KAM and GND density, such as in G2 and G3. Consequently, the formation of substructures and the emergence of pronounced orientation scattering are observed within the grains. Pronounced KAM values and GND densities are also observed at the austenite–martensite interfaces. This phenomenon is driven by the significant strength gradient across the interface. The softer austenite phase undergoes substantially larger plastic deformation than the adjacent harder martensite. To accommodate this strain mismatch and maintain deformation compatibility between the two phases, a substantial accumulation of GNDs is necessary at the interface. The increase in GND density also contributes to strain hardening by refining the mean free path for dislocation movement.

### 3.4. Strain-Induced Twinning and Martensite Transformation

**Figure 6:**
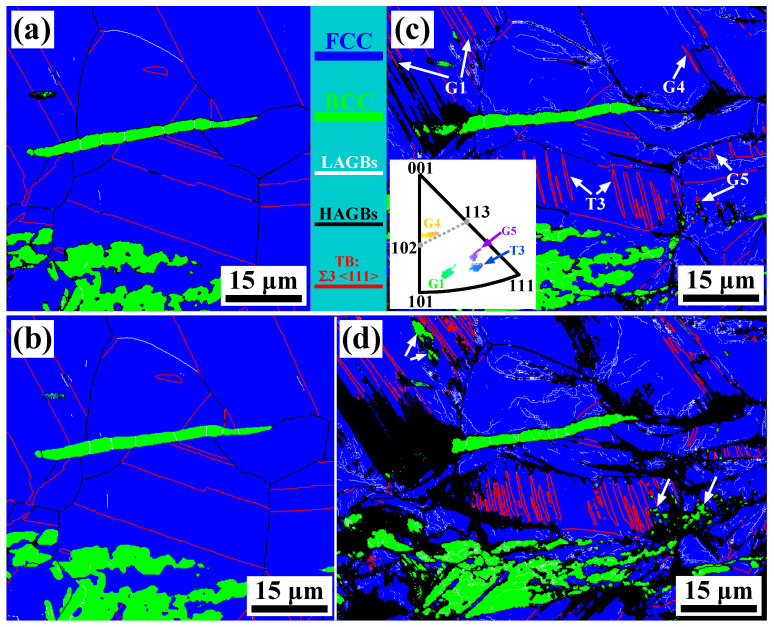
EBSD phase maps of the randomly selected region at different stages of deformation: (**a**) initial state, (**b**) first step, (**c**) second step, and (**d**) third step.

The microstructural evolution presented in Figure 6 characterizes grain boundary development and phase transformation, employing the following color scheme: white lines for low-angle grain boundaries (LAGBs, 2–10°), black lines for high-angle grain boundaries (HAGBs, >10°), and red lines for TBs. The blue and green regions represent the FCC and BCC phases, respectively. As can be observed in Figure 6c, the boundaries undergo marked changes in the second deformation step, with both a rise in LAGBs and the formation of TBs. LAGBs develop preferentially at grain boundaries, austenite–martensite interfaces, and slip bands, with some even extending across entire grains to form enclosed structures. The progressive accumulation of misorientation in some LAGBs facilitates their transformation from LAGBs to HAGBs. Concurrent with the degradation of pre-existing TBs, newly formed mechanical TBs are observed within certain grains. The observed mechanical twins are parallel to the slip traces, confirming that dislocation activity is a prerequisite for twinning. The formation of mechanical twins exhibits strong dependence on the initial orientation of the FCC grains. As shown in the inset of Figure 6c, mechanical twins preferentially develop in grains with initial orientations located below the <113>–<102> connecting line in the IPF.

The formation of deformation twins is closely related to the glide of like-signed Shockley partial dislocations on successive {111} planes [29,42,43]. During plastic deformation, a perfect (110) dislocation is prone to dissociating into two Shockley partials, separated by a ribbon of intrinsic stacking fault. The degree of separation between these two mobile Shockley partials serves as a key microstructural parameter influencing twin embryo stability, with large separation promoting successful twinning formation. This separation distance between the two partial dislocations, known as the stacking fault width, is larger in materials with a low SFE than in those with a high SFE [44]. The high stress required to compress stacking faults makes cross-slip difficult in low-SFE materials, thereby promoting planar slip. If the planar slip of partial dislocations leads to the widening and overlapping of stacking faults on adjacent slip planes, mechanical twinning may initiate at these sites. For the grains with initial orientations located below the <113>–<102> connecting line, the leading Shockley partial experiences higher shear stress than the trailing one [45]. By driving the separation of Shockley partials, this stress gradient enhances the overlapping of stacking faults on adjacent slip planes, thus facilitating mechanical twin formation.

Newly formed martensite is also observed in the deformed microstructure, as indicated by the arrows in Figure 6c,d. This martensite preferentially forms within the mechanical twins, demonstrating that the transformation sequence of austenite → mechanical twinning → martensite is predominant for the tested material in this study. A similar sequence of strain-induced martensite transformation has been reported in AISI 304 steel during cold drawing [46]. Nevertheless, the strain-induced martensite formed in this study exhibits an extremely low volume fraction of merely ~0.5 vol.%, as shown in Figure 6d. Other potential nucleation sites cannot be completely excluded based on current observations. In addition, it is reported that low-Ni (~8 wt.%) 304 stainless steel preferentially produces strain-induced martensite instead of deformation twins under tensile deformation conditions [25], which differs from the results reported in this work. The specific mechanism behind this discrepancy remains unclear based on our current available knowledge.

The above analysis reveals the simultaneous activity of dislocation slip, mechanical twinning, and martensite transformation in this work. Continuous lattice rotation and the rising GND density confirm that dislocation slip remains the dominant deformation mechanism across all hardening stages. Dense dislocation entanglements block dislocation glide and provide a fundamental mechanism for strain hardening. Mechanical twinning simultaneously facilitates plastic deformation and induces remarkable strengthening. It is reported that mechanical twinning can facilitate plastic deformation both by introducing additional slip systems and by promoting stress redistribution among existing systems via dislocation–twin interactions [44]. TBs not only act as barriers to specific gliding dislocations but also serve as sinks for them, causing dislocation pile-ups. This behavior reduces the mean free path for dislocation motion and enhances strain hardening through a dynamic Hall–Petch effect. Furthermore, the strain-induced martensite also contributes to the strain hardening due to its relatively higher strength. Nevertheless, owing to the limited amount of strain-induced martensite observed from microstructural characterization, its contribution to hardening is inferior to that of mechanical twinning. Therefore, the strain hardening behaviors in stage B and C shown in Figure 2d should be attributed to the combined hardening effect of mechanical twins and strain-induced martensite.

## 4. Conclusions

(1) The serviced 304 stainless steel contains 90 vol.% austenite and 10 vol.% pre-existing martensite. It has an ultimate tensile strength of 652 MPa and a fracture elongation of 83.4%, with strain hardening separated into three distinct stages. Stage A is controlled by dislocation slip and dynamic recovery. Stages B and C depend on the coupled strengthening of mechanical twinning and strain-induced martensite. Grain deformation is heterogeneous and dominated by slip systems with the top two Schmid factors.

(2) Deformation mechanisms evolve sequentially from dislocation slip to mechanical twinning and then strain-induced martensite transformation. Mechanical twins serve as the preferential nucleation sites for strain-induced martensite. Only limited newly formed martensite is observed in deformed microstructures. Thus, mechanical twinning, instead of martensite transformation, dominates the secondary deformation.

(3) This quasi in situ EBSD study systematically reveals the complete tensile deformation and strain hardening sequence of serviced 304 stainless steel. The identified deformation mechanisms provide an experimental basis on which to evaluate residual ductility and failure risks for serviced austenitic stainless steel components.

## Figures and Tables

**Table 1:** The four slip systems with the top Schmid factors for certain grains.

Grains ID	Slip Systems		Schmid Factors
G1	S1	($\bar{1}11$)[$\bar{1}0\bar{1}$]	0.470
	S2	($\bar{1}1\bar{1}$)[$\bar{1}01$]	0.384
	S3	($\bar{1}11$)[$01\bar{1}$]	0.382
	S4	($\bar{1}1\bar{1}$)[011]	0.270
T1	S1	($1\bar{1}\bar{1}$)[$0\bar{1}1$]	0.474
	S2	($1\bar{1}1$)[$0\bar{1}\bar{1}$]	0.469
	S3	($\bar{1}\bar{1}1$)[$1\bar{1}0$]	0.388
	S4	($1\bar{1}1$)[$\bar{1}\bar{1}0$]	0.383
G2	S1	($\bar{1}1\bar{1}$)[$\bar{1}01$]	0.493
	S2	($\bar{1}11$)[$\bar{1}0\bar{1}$]	0.481
	S3	($\bar{1}1\bar{1}$)[011]	0.269
	S4	($\bar{1}11$)[$01\bar{1}$]	0.247
G3	S1	($11\bar{1}$)[$\bar{1}10$]	0.495
	S2	($\bar{1}1\bar{1}$)[110]	0.488
	S3	($\bar{1}11$)[$01\bar{1}$]	0.271
	S4	($\bar{1}1\bar{1}$)[011]	0.264
T3	S1	($\bar{1}\bar{1}\bar{1}$)[$0\bar{1}1$]	0.432
	S2	($\bar{1}\bar{1}\bar{1}$)[$\bar{1}01$]	0.359
	S3	($1\bar{1}1$)[$\bar{1}\bar{1}0$]	0.285
	S4	($\bar{1}\bar{1}1$)[$0\bar{1}\bar{1}$]	0.271

## Data Availability

The original contributions presented in this study are included in the article. Further inquiries can be directed to the corresponding authors.
